# 6-Nitrodopamine Is the Most Potent Endogenous Positive Inotropic Agent in the Isolated Rat Heart

**DOI:** 10.3390/life13102012

**Published:** 2023-10-04

**Authors:** José Britto-Júnior, Lincoln Rangel Medeiros-Teixeira, Antonio Tiago Lima, Letícia Costa Dassow, Rodrigo Álvaro Brandão Lopes-Martins, Rafael Campos, Manoel Odorico Moraes, Maria Elisabete A. Moraes, Edson Antunes, Gilberto De Nucci

**Affiliations:** 1 Department of Pharmacology, Faculty of Medical Sciences, University of Campinas (UNICAMP), Campinas 13083-970, Brazil; josebrittojr@dac.unicamp.br (J.B.-J.); l219199@dac.unicamp.br (L.R.M.-T.); a234935@dac.unicamp.br (A.T.L.); racampos@unicamp.br (R.C.); eantunes@unicamp.br (E.A.); 2 Laboratory of Biophotonics and Experimental Therapeutics, University Evangélica of Goiás (UniEVANGÉLICA), Anápolis 75083-515, Brazil; llcdassow@gmail.com (L.C.D.); ralopesmartins@gmail.com (R.Á.B.L.-M.); 3 Clinical Pharmacology Unit, Drug Research and Development Center, Federal University of Ceará (UFC), Fortaleza 60020-181, Brazil; odorico@ufc.br (M.O.M.); betemora@ufc.br (M.E.A.M.); 4 Department of Pharmacology, Institute of Biomedical Sciences, University of São Paulo (USP), Sāo Paulo 05508-220, Brazil

**Keywords:** atenolol, b-blocker, tetrodotoxin, L-NAME

## Abstract

Background: 6-nitrodopamine released from rat isolated atria exerts positive chronotropic action, being more potent than noradrenaline, adrenaline, and dopamine. Here, we determined whether 6-nitrodopamine is released from rat isolated ventricles (RIV) and modulates heart inotropism. Methods: Catecholamines released from RIV were quantified by LC-MS/MS and their effects on heart inotropism were evaluated by measuring left ventricular developed pressure (LVDP) in Langendorff’s preparation. Results: 6-nitrodopamine was the major released catecholamine from RIV. Incubation with L-NAME (100 µM), but not with tetrodotoxin (1 µM), caused a significant reduction in 6-nitrodopamine basal release. 6-nitrodopamine release was significantly reduced in ventricles obtained from L-NAME chronically treated animals. 6-nitrodopamine (0.01 pmol) caused significant increases in LVDP and dP/dt_max_, whereas dopamine and noradrenaline required 10 pmol, and adrenaline required 100 pmol, to induce similar increases in LVDP and dP/dt_max_. The infusion of atenolol (10 nM) reduced basal LVDP and blocked the increases in LVDP induced by 6-ND (0.01 pmol), without affecting the increases in LVDP induced by 10 nmol of dopamine and noradrenaline and that induced by adrenaline (100 nmol). Conclusions: 6-nitrodopamine is the major catecholamine released from rat isolated ventricles. It is 1000 times more potent than dopamine and noradrenaline and is selectively blocked by atenolol, indicating that 6-ND is a main regulator of heart inotropism.

## 1. Introduction

Myocardial inotropism and lusitropism are modulated by the β-adrenoceptors (b-AR) β_1_-AR and β_2_-AR, which are mainly located in human cardiomyocytes (β_1_-/β_2_-AR ratio is 80%:20%) [1]. A higher preponderance of β_1_-AR over β_2_-AR has also been observed in rat right and left atria (67–33%, respectively) [2] and in the rat left ventricle [3]. The β_3_-adrenoceptors are also expressed in the heart [4], but they are associated with a negative inotropic response [5].

The catecholamines noradrenaline and adrenaline are known as potent positive chronotropic and inotropic agents through β-AR activation [6]. When these receptors are activated, they cause intracellular signaling via G protein modulation of adenylyl cyclases, a family of enzymes responsible for cAMP production [7]. β_1_ agonists such as dobutamine increase tissue cAMP production through the β-adrenergic-mediated stimulation of adenylate cyclase [8]. The current understanding is that under basal conditions, cAMP levels are modulated by either intrinsic β-AR activities or by classical catecholamine baseline levels. Interestingly, cAMP is rapidly hydrolyzed by phosphodiesterases (PDEs) and the latter plays an important part in directing intracellular cAMP propagation [9,10]. Indeed, the use of selective phosphodiesterase (PDE) type 3 inotropes such as milrinone [11] or enoximone [12] provokes indirect increases in cAMP production, and the inhibition of PDE3 or PDE4 augments the noradrenaline-induced positive inotropic effects in the human atrium [13].

The novel catecholamine 6-nitrodopamine (6-ND) is released from the human umbilical cord artery and vein [14], aortae obtained from the tortoise *Chelonoidis carbonarius* [15] and the marmoset *Callithrix* spp. [16], and human [17] and rat vas deferens [18] and right atria [19]. In the atria, 6-ND acts as a potent modulator of heart chronotropism, being more potent than noradrenaline and adrenaline (100×), and dopamine (10,000×) [19]. Incubation of the atria with the nitric oxide (NO) synthase inhibitor L-NAME causes a significant reduction in 6-ND basal released levels, whereas incubation with the voltage-gated sodium channel blocker tetrodotoxin does not affect it, indicating that in contrast to noradrenaline, the origin of 6-ND is not neurogenic [19]. Indeed, the release of 6-ND from mice isolated atria and ventricles was significantly reduced in eNOS^−/−^, but not in nNOS^−/−^ or iNOS^−/−^ mice [20]. Another characteristic of the 6-ND chronotropic effect in the rat isolated atria is that the 6-ND-induced positive chronotropic effect was selectively blocked by the β_1_-adrenoceptor antagonists atenolol, betaxolol, and metoprolol, since the concentrations employed had no effect on the chronotropism increases provoked by the classical catecholamines [19].

Since noradrenaline and adrenaline are considered potent stimulators of heart inotropism [21], whether 6-ND is released from rat isolated ventricles and whether it modulates heart inotropism in Langendorff’s preparation was investigated [22].

## 2. Materials and Methods

### 2.1. Animals

The animals (male Wistar rats, weighing 280–320 g) were acquired from CEMIB-UNICAMP (São Paulo, Brazil). The protocols were approved by the local ethics committee (CEUA; Protocol No. 5746-1/2021; 5831-1/2021) according to the Brazilian Guidelines (CONCEA) [23]; and the ARRIVE guidelines [24].

### 2.2. Treatment with NO Synthesis Inhibitor N^ω^-Nitro-L-arginine Methyl Ester Hydrochloride (L-NAME)

Animals were given filtered water containing L-NAME at a dose of approximately 20 mg/rat/day for a minimum of 4 weeks and a maximum of 6 weeks [25]. Control animals were treated with filtered water.

### 2.3. Rat Isolated Ventricles

Isoflurane overdose was employed for euthanasia and the animals were exposed to a concentration greater than 5% until 1 min after breathing stopped. Exsanguination was performed to confirm the euthanasia. After euthanasia, the chest was opened and the heart was rapidly excised. The right and left ventricles were isolated en bloc from each rat heart and suspended in a 5 mL organ bath containing Krebs–Henseleit solution (KHS; in mM: NaCl 118, KCl 4.7, CaCl_2_ 2.5, MgSO_4_ 1.7, NaHCO_3_ 24.9, KH_2_PO_4_ 1.2, dextrose 11, sodium pyruvate 2, pH 7.4) continuously gassed with a mixture (95% O_2_: 5% CO_2_) at 37 °C, supplemented with ascorbic acid (1 mM) to prevent catecholamine oxidation [26].

### 2.4. Basal Release of Catecholamines from Rat Ventricles

The right and left ventricles en bloc from each rat were suspended in a 5 mL organ bath containing KHS (pH 7.4; 95% O_2_ / 5% CO_2_; 37 °C) containing ascorbic acid (1 mM) for 30 min. Two 2 mL aliquots of the supernatant were transferred to black Eppendorf tubes and stored at −20 °C until analysis [27]. Basal release of catecholamines was also evaluated from ventricles obtained from animals chronically treated with L-NAME and from ventricles of control animals incubated with either L-NAME (100 µM) or the voltage-gated sodium channel blocker tetrodotoxin (TTX; 1 µM).

### 2.5. Determination of Catecholamines by Liquid Chromatography Coupled to Tandem Mass Spectrometry (LC-MS/MS)

The catecholamine LC-MS/MS method [28] was modified to allow the measurement of the four catecholamines in a single chromatographic run. The method validation following USFDA guidelines for bioanalytical methods [29] was described elsewhere.

### 2.6. Isolated Langendorff’s Perfused Heart

Heparin (1000 IU/kg) was injected i.p. to prevent blood clotting and euthanasia was performed as described earlier (Section 2.3). Exsanguination was performed to confirm the euthanasia. The chest was opened and the heart was rapidly excised, the ascending aorta was cannulated, and the heart was mounted in a nonrecirculating Langendorff apparatus. The isolated heart was perfused with KHS (pH 7.4, 37 °C), equilibrated with carbogen gas mixture (95% O_2_: 5% CO_2_) at constant flow (10 mL/min) and maintained 4-10 mm Hg left ventricular end-diastolic pressure (LVEDP) during initial equilibration [30]. A water-filled latex balloon, connected to the pressure transducer (MLT1199 BP Transducer, ADInstruments, Inc., Dunedin, NZ, USA), was inserted into the left ventricle (LV) via the mitral valve. Left ventricular systolic pressure (LVSP), left ventricular end-diastolic pressure (LVeDP), and heart rate (HR) were continuously recorded by a PowerLab System (ADInstruments, Inc., Dunedin, NZ, USA). Left ventricular developed pressure (LVDP) was calculated by the following formula: LVSP-LVeDP and expressed in mmHg. Rate pressure product (RPP) was calculated as the product of heart rate (HR) and left ventricular developed pressure (LVDP) as follows: RPP = (HR × LVDP). One heart was used for a single drug and for a single dose. Only hearts that presented a basal heart rate between 250 and 300 bpm were employed in the experiments.

### 2.7. Langendorff’s Perfused Heart Analysis and Experimental Design

Following an equilibration period of at least ten minutes, a single bolus (10 mL) of 6-ND (0.01, 0.1, 1 or 10 pmol), dopamine (1, 10, 100 or 1000 pmol), noradrenaline (1, 10, 100 or 1000 pmol), or adrenaline (1, 10, 100 or 1000 pmol) was injected. One heart was used for a single drug and for a single dose (for the evaluation of the inotropic effect of 0.01 (*n* = 5), 0.1 (*n* = 6), 1 (*n* = 6), and 10 (*n* = 6) pmol of 6-ND, 23 animals were employed).

### 2.8. Effect of Atenolol Infusion on the Positive Inotropic Effect Induced by Catecholamines

Following an equilibration period of at least ten minutes, a ten-minute (100 mL/min) infusion of atenolol (0.001, 0.01, 0.1 and 1 µM; final concentration) was performed before the bolus injection of 6-ND (1 pmol), dopamine (10 nmol), noradrenaline (1 nmol), and adrenaline (1 nmol). The heart was monitored for 15 min. One heart was used for a single drug and a single infusion.

### 2.9. Statistical Analysis

The left ventricular developed pressure (LVDP) was calculated as the difference between the systolic and end-diastolic pressure values, received from the LVDP curve. Left ventricular systolic pressure (LVSP), left ventricular end-diastolic pressure (LVeDP), and heart rate (HR) were continuously recorded by a PowerLab System (ADInstruments, Inc., Dunedin, NZ, USA). Left ventricular developed pressure (LVDP) was calculated by the following formula: LVSP-LVeDP and expressed in mmHg. Rate pressure product (RPP) was calculated by the following formula: HR times LVDP and expressed as beats × mmHg × min^−1^. The maximal rate of rise of the left ventricular pressure (+dP/dt_max_) was monitored continuously by a pressure transducer connected to a PowerLab system (AD Instrument, Dunedin, New Zealand) and expressed as mmHg × s^−1^. Changes in LVDP were expressed as the increase above the baseline. Data of the heart rate are presented as beats per minute (bpm). The results are presented as the mean ± standard error of the mean (SEM). Paired and unpaired *t*-tests were used when appropriate. Comparisons among three or more groups were evaluated using one-way ANOVA, followed by Newman–Keuls test. Actual *p* values are described in Figures.

### 2.10. Chemical and Reagents

The reagents and suppliers are described in Table 1 below.

## 3. Results

### 3.1. Basal Release of Catecholamines from Isolated Ventricles

**Figure 1:**
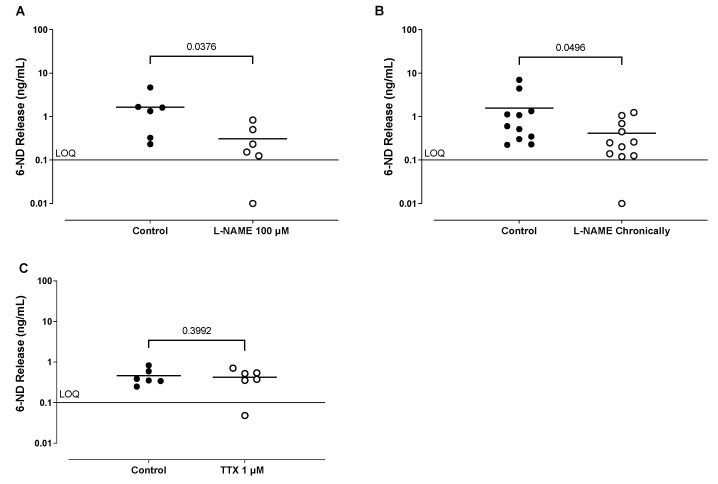
Basal release of 6-nitrodopamine (6-ND) from rat isolated ventricles. Panel (**A**) shows the effect of pre-incubation (30 min) with L-NAME (100 µM) on the basal release of 6-ND from rat isolated ventricles (*n* = 6). Panel (**B**) shows the basal release of 6-ND from isolated ventricles obtained from control rats and from chronically L-NAME-treated rats (20 mg/kg/day, 4–6 weeks, *n* = 11). Panel (**C**) shows effect of pre-incubation (30 min) of tetrodotoxin (TTX;1 µM) on the basal release of 6-ND (*n* = 6).

The isolated ventricles (Figure 1A–C) presented 6-ND basal release, which was significantly inhibited by pre-treating (30 min) the isolated ventricles with L-NAME (100 µM; Figure 1A, *n* = 6). The basal release of 6-ND was also decreased in the ventricles obtained from rats chronically treated with L-NAME (Figure 1B, *n* = 11). The incubation of rat isolated ventricles with TTX (30 min, 1 µM) had no effect on the 6-ND basal release (Figure 1C, *n* = 6). Dopamine, noradrenaline, and adrenaline levels were undetected in all samples (limit of quantitation was 0.1 ng/mL).

### 3.2. Effect of the Chronic Administration of L-NAME on the Left Ventricular Developed Pressure (LVDP), dP/dt_max_, Heart Rate (HR), and Rate Pressure Product (RPP)

**Figure 2:**
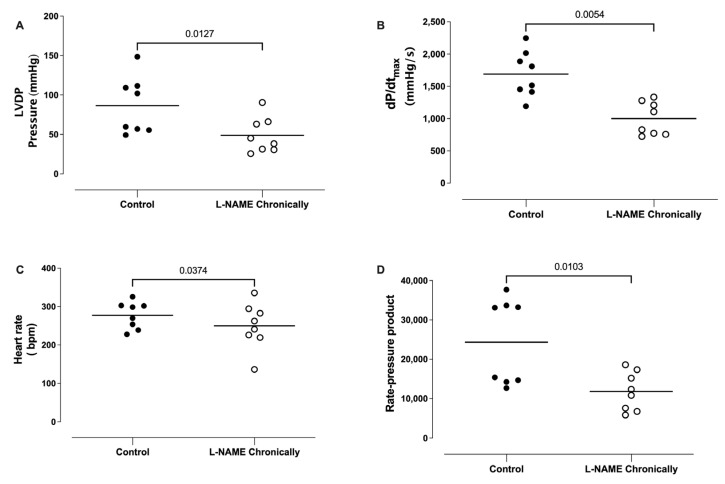
Effect of the chronic treatment with L-NAME on the left ventricular pressure (LVDP), maximal rate of rise of left ventricular pressure (dP/dt_max_), heart rate, and rate pressure product (RPP). The animals were treated with L-NAME (20 mg/day for 4–6 weeks), and the hearts isolated and perfused in vitro (Langendorff’s preparation). The hearts obtained from chronically L-NAME-treated animals presented significantly lower left ventricular developed pressure (Panel **A**), lower maximal rate of rise of left ventricular pressure (dP/dt_max_; Panel **B**), lower heart rate (Panel **C**), and lower rate-pressure product (Panel **D**), compared to hearts obtained from control animals. Unpaired *t*-test.

The animals were chronically (4-6 weeks) treated with L-NAME (20 mg/day) in the drinking water, and the hearts were isolated and perfused in vitro (Langendorff’s preparation). The hearts obtained from the chronically L-NAME-treated animals presented a significantly lower left ventricular developed pressure (Figure 2A), lower maximal rate of rise of left ventricular pressure (dP/dt_max_; Figure 2B), lower heart rate (Figure 2C), and lower rate-pressure product (Figure 2D), compared to hearts obtained from the control animals.

### 3.3. Effect of Bolus Injections of Catecholamines on the Left Ventricle Developed Pressure (LVDP)

**Figure 3:**
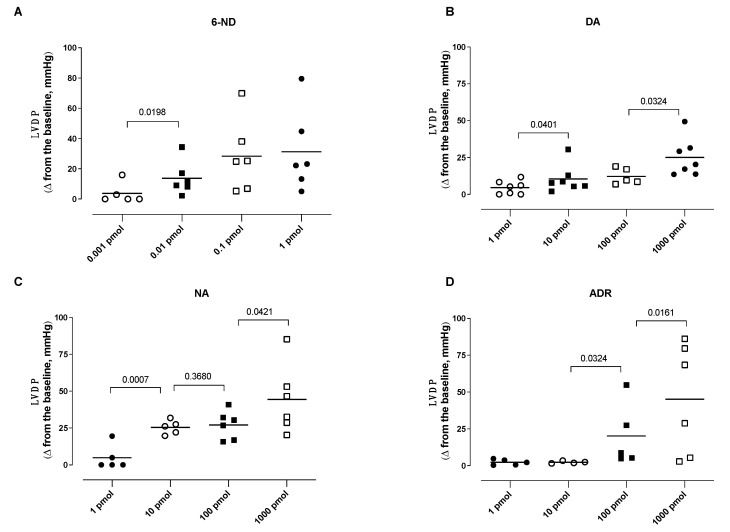
Effect of bolus injections of 6-nitrodopamine (6-ND), dopamine (DA), noradrenaline (NA), and adrenaline (ADR) on the left ventricular developed pressure (LVDP). Bolus injections of 6-ND (0.01–1 pmol; Panel **A**), dopamine (10–1000 pmol; Panel **B**), noradrenaline (10–1000 pmol; Panel **C**), and adrenaline (100–1000 pmol; Panel **D**) caused significant increases in the left ventricular developed pressure (LVDP). ANOVA, followed by Newman–Keuls test.

A bolus injection of 6-ND (0.01 pmol) caused a significant increase in the LVDP (Figure 3A). Further increases in the doses of 6-ND (0.1 and 1 pmol) also substantially increased LVDP, but these increases were not significantly different among them (Figure 3A). In contrast to 6-ND, bolus injections (1 pmol) of dopamine (Figure 3B), noradrenaline (Figure 3C), and adrenaline (Figure 3D) caused no increases in LVDP. Bolus injections of higher doses (10–1000 pmol) of dopamine (Figure 3B) and noradrenaline (Figure 3C) caused significant dose-dependent increases in the LVDP. A bolus injection of higher doses (100–1000 pmol) of adrenaline (Figure 3D) caused significant dose-dependent increases in the LVDP.

### 3.4. Effect of Bolus Injections of Catecholamines on dP/dt_max_

**Figure 4:**
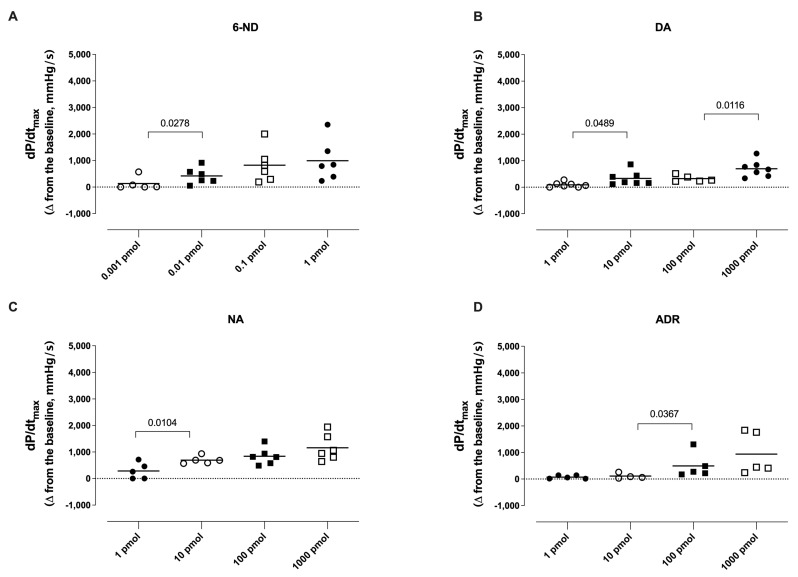
Effect of bolus injections of 6-nitrodopamine (6-ND), dopamine (DA), noradrenaline (NA), and adrenaline (ADR) on the maximal rate of rise of left ventricular pressure (dP/dt_max_). Bolus injections of 6-ND (0.01–1 pmol; Panel **A**), dopamine (10–1000 pmol; Panel **B**), noradrenaline (10–1000 pmol; Panel **C**), and adrenaline (100–1000 pmol; Panel **D**) caused significant increases in the maximal rate of rise of left ventricular pressure (dP/dt_max_). ANOVA, followed by Newman–Keuls test.

A bolus injection of 6-ND (0.01 pmol) caused a significant increase in the dP/dt_max_ (Figure 4A). Further increases in the doses of 6-ND also substantially increased dP/dt_max_, but these increases were not significantly different among them (Figure 4A). In contrast to 6-ND, bolus injections (1 pmol) of dopamine (Figure 4B), noradrenaline (Figure 4C), and adrenaline (Figure 4D) caused no increases in the dP/dt_max_. Bolus injections of higher doses of dopamine (Figure 4B), noradrenaline (Figure 4C), and adrenaline (Figure 4D) caused significant dose-dependent increases in the dP/dt_max_.

### 3.5. Effect of Bolus Injections of Catecholamines on the Heart Rate

**Figure 5:**
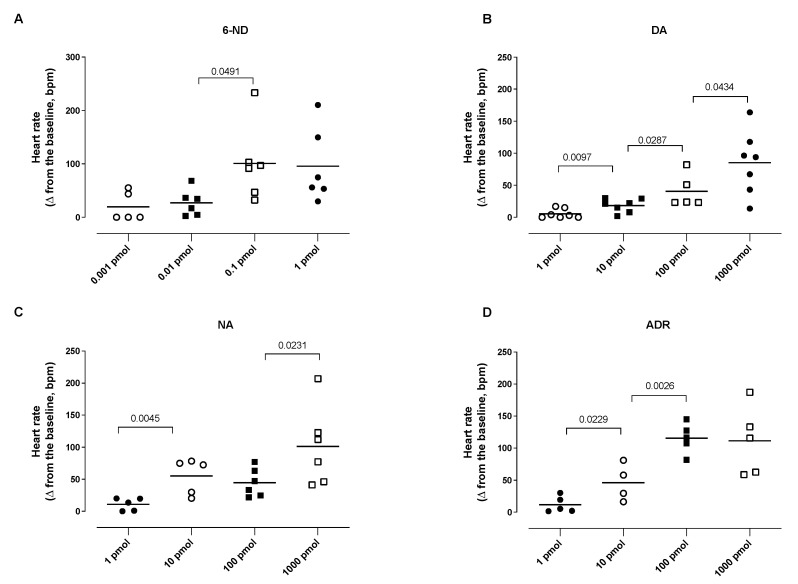
Effect of bolus injections of 6-nitrodopamine (6-ND), dopamine (DA), noradrenaline (NA), and adrenaline (ADR) on the heart rate (HR). Bolus injections of 6-ND (0.1–1 pmol; Panel **A**), dopamine (10–1000 pmol; Panel **B**), noradrenaline (10–1000 pmol; Panel **C**), and adrenaline (10–1000 pmol; Panel **D**) caused significant increases in the heart rate. ANOVA, followed by Newman–Keuls test.

A bolus injection of 6-ND (0.1 pmol) caused a significant increase in the heart rate (Figure 5A). Further increases in the doses of 6-ND also substantially increased the heart rate, but these increases were not significantly different among them (Figure 5A). In contrast to 6-ND, bolus injections (1 pmol) of dopamine (Figure 5B), noradrenaline (Figure 5C), and adrenaline (Figure 5D) caused no increases in the heart rate. Bolus injections of higher doses (10-1000 pmol) of dopamine (Figure 5B), noradrenaline (Figure 5C), and adrenaline (Figure 5D) caused significant dose-dependent increases in the heart rate.

### 3.6. Effect of Atenolol Infusion on the Increases in the Left Ventricle Developed Pressure (LVDP) Induced by Catecholamines

**Figure 6:**
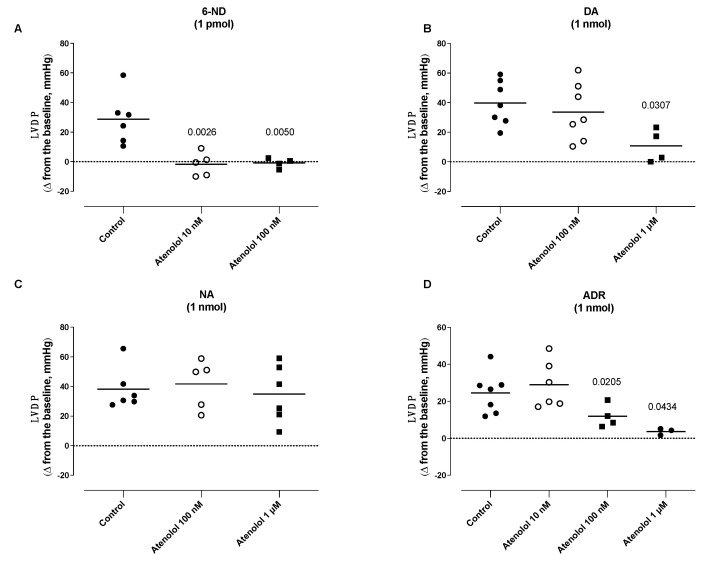
Effect of atenolol infusions (0.01–1 µM) on the increases in the left ventricle developed pressure (LVDP) induced by 6-nitrodopamine (6-ND), dopamine (DA), noradrenaline (NA), and adrenaline (ADR). Atenolol (10–100 nM) blocked the increases in the LVDP induced by 6-ND (1 pmol; Panel **A**). Atenolol (100 nM) had no effect on the increases in the LVDP induced by dopamine (1 nmol; Panel **B**) and noradrenaline (1 nmol; Panel **C**), but significantly inhibited the increases in the LVDP induced by adrenaline (1 nmol; Panel **D**). Atenolol (1 µM) significantly reduced the increases in the LVDP induced by dopamine (1 nmol; Panel **B**), noradrenaline (1 nmol; Panel **C**), and adrenaline (1 nmol; Panel **D**). ANOVA, followed by Newman–Keuls test. The *p* values reflect significance compared to control values.

The infusion of atenolol (10 nM and 100 nM) almost abolished the increases in the LVDP induced by 1 pmol of 6-ND (Figure 6A). Atenolol (100 nM) significantly reduced the increases in the LVDP induced by adrenaline (1 nmol; Figure 6D), without affecting those induced by either dopamine (1 nmol; Figure 6B) or noradrenaline (Figure 6C). The infusion of atenolol (1 µM) significantly reduced the increases in the LVDP induced by dopamine (1 nmol; Figure 6B), noradrenaline (1 nmol; Figure 6C), and adrenaline (1 nmol; Figure 6D).

### 3.7. Effect of Atenolol Infusion on the Increases in dP/dt_max_ Induced by Catecholamines

**Figure 7:**
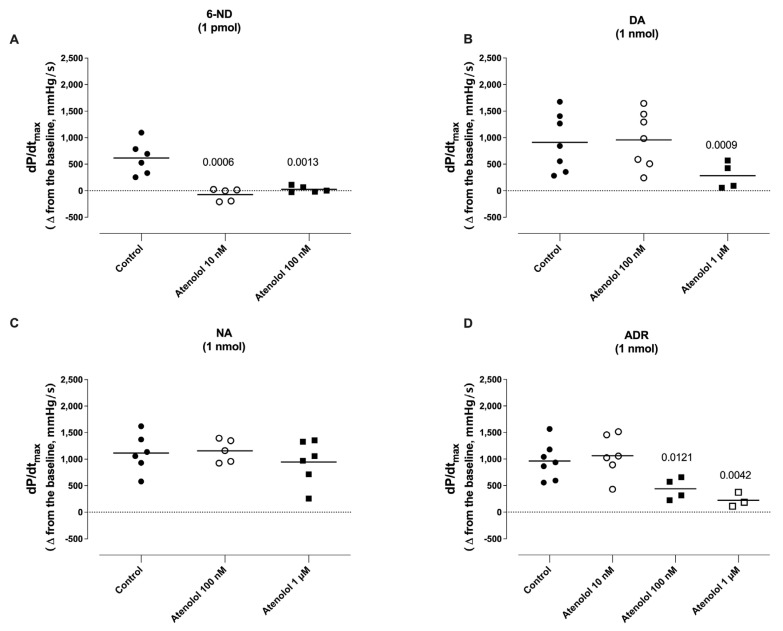
Effect of atenolol infusions (0.01–1 nM) on the maximal rate of rise of left ventricular pressure (dP/dt_max_) induced by 6-nitrodopamine (6-ND), dopamine (DA), noradrenaline (NA), and adrenaline (ADR). Atenolol (10–100 nM) blocked the increases in the dP/dt_max_ induced by 6-ND (1 pmol; Panel **A**). Atenolol (100 nM) had no effect on the increases in the dP/dt_max_ induced by dopamine (1 nmol; Panel **B**) and noradrenaline (1 nmol; Panel **C**), but significantly inhibited the increases in the dP/dt_max_ induced by adrenaline (1 nmol; Panel **D**). Atenolol (1 µM) significantly reduced the increases in the dP/dt_max_ induced by dopamine (1 nmol; Panel **B**), noradrenaline (1 nmol; Panel **C**), and adrenaline (1 nmol; Panel **D**). ANOVA, followed by Newman–Keuls test. The *p* values reflect significance compared to control values.

The infusion of atenolol (10 nM and 100 nM) almost abolished the increases in the dP/dt_max_ induced by 1 pmol of 6-ND (Figure 7A). The infusion of atenolol (100 nM) significantly reduced the increases in the dP/dt_max_ induced by adrenaline (1 nmol; Figure 7D), without affecting those induced by either dopamine (1 nmol; Figure 7B) or noradrenaline (Figure 7C). The infusion of atenolol (1 µM) significantly reduced the increases in the dP/dt_max_ induced by dopamine (1 nmol; Figure 7B), noradrenaline (1 nmol; Figure 7C), and adrenaline (1 nmol; Figure 7D).

### 3.8. Effect of Atenolol Infusion on the Increases in the Heart Rate (HR) Induced by Catecholamines

**Figure 8:**
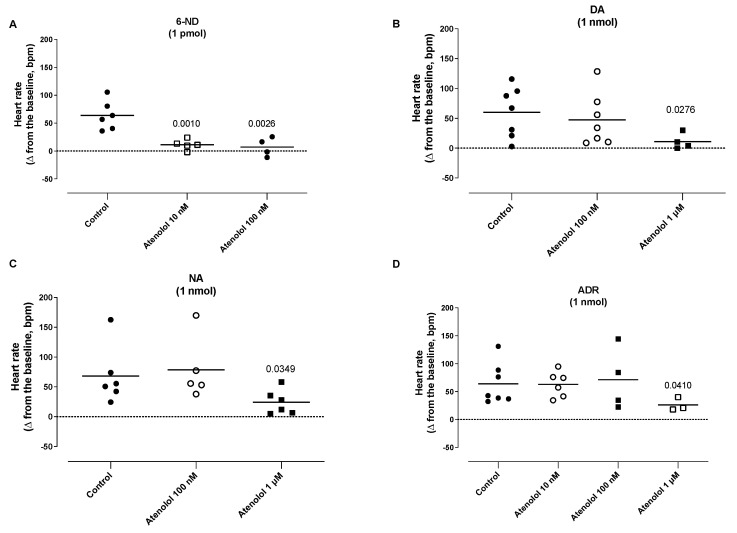
Effect of atenolol infusions (0.01–1 µM) on the increases in heart rate induced by 6-nitrodopamine (6-ND), dopamine (DA), noradrenaline (NA), and adrenaline (ADR). Atenolol (10–100 nM) blocked the increases in the heart rate induced by 6-ND (1 pmol; Panel **A**). Atenolol (100 nM) had no effect on the increases in the heart rate induced by dopamine (1 nmol; Panel **B**), noradrenaline (1 nmol; Panel **C**), and adrenaline (1 nmol; Panel **D**). Atenolol (1 µM) caused significant reductions in the increases in the heart rate induced by dopamine (1 nmol; Panel **B**), noradrenaline (1 nmol; Panel **C**), and adrenaline (1 nmol; Panel **D**). ANOVA, followed by Newman–Keuls test. The *p* values reflect significance compared to control values.

The infusion of atenolol (10 nM and 100 nM) significantly attenuated the increases in the heart rate induced by 1 pmol of 6-ND (Figure 8A). The infusion of atenolol (100 nM) did not alter the increases in the HR induced by dopamine (1 nmol; Figure 8B), noradrenaline (1 nmol; Figure 8C), and adrenaline (Figure 8D). The infusion of atenolol (1 µM) attenuated the increases in the heart rate induced by dopamine (1 nmol; Figure 8B), noradrenaline (1 nmol; Figure 8C), and adrenaline (Figure 8D).

### 3.9. Effect of Atenolol Infusion on the Basal LVDP, dP/dt_max_, HR, and Rate Pressure Product

**Figure 9:**
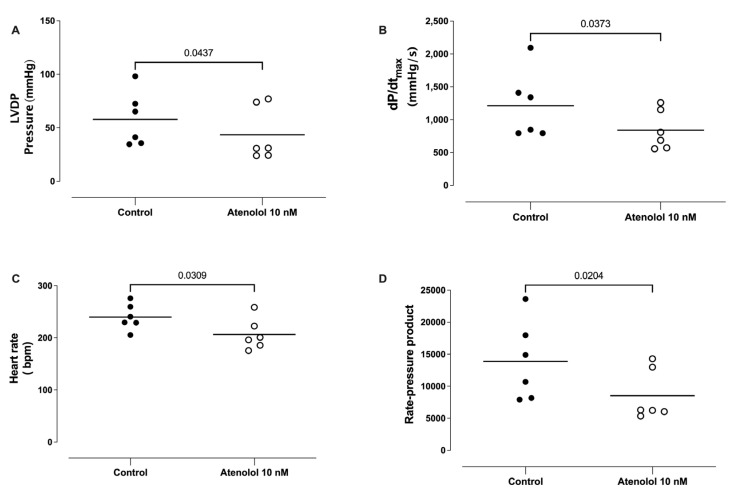
Effect of atenolol on the left ventricular pressure (LVDP), maximal rate of rise of left ventricular pressure (dP/dt_max_), heart rate, and rate pressure product (RPP). Atenolol (10 nM) caused significant reductions in the left ventricular developed pressure (LVDP; Panel **A**), maximal rate of rise of left ventricular pressure (dP/dt_max_; Panel **B**), heart rate (Panel **C**), and rate-pressure product (Panel **D**). Unpaired *t*-test.

The infusion of atenolol (10 nM) caused significant reductions in the LVDP (Figure 9A), the dP/dt_max_ (Figure 9B), the basal heart rate (Figure 9C), and the basal rate pressure product (Figure 9D).

## 4. Discussion

6-ND is released from the whole heart; as it is not only the main catecholamine released from rat isolated ventricles, the results presented here extend our original observation that 6-ND is the major catecholamine released from rat isolated atria [19]. As observed in vascular tissues [14,15], rat and human vas deferens [17,18] and rat isolated atria [19], the basal release is significantly reduced when the tissues are pre-incubated with the NO synthase inhibitor L-NAME; or, in the case of the rat vas deferens, rat isolated atria and rat isolated ventricles (present study) when tissues from L-NAME-chronically-treated rats were employed. 6-ND biosynthesis could be due to either direct nitrosation of dopamine following NO synthesis or to nitrite anion (NO_2_^−^) oxidation, formed by the degradation of NO to the nitrogen dioxide radical (NO_2_^.^), as observed with mammalian heme peroxidases [31] and myeloperoxidase [32]. The finding that 6-ND basal release is significantly decreased in mice isolated atria and ventricles obtained from eNOS^−/−^ mice, but is not reduced in nNOS^−/−^ mouse [20], indicates a non-neurogenic origin in the heart.

The results with Langendorff’s preparation demonstrate that 6-ND is a very potent positive inotropic agent, being more potent than dopamine and noradrenaline (one thousand times) and adrenaline (ten thousand times). 6-ND should be considered the most potent endogenous inotropic agent ever reported. Since the synthesis/release of 6-ND from the ventricles is significantly reduced following both acute and chronic inhibition of NO synthase, the finding that rat hearts from chronically L-NAME-treated rats present decreased LVDP is compatible with the major physiological role of 6-ND as a modulator of heart inotropy; however, those hearts present extensive areas of fibrosis and myocardial necrosis [33], which could contribute to the decreased inotropic response.

The inhibition of NO synthase does cause coronary vasoconstriction [34], and coronary vasoconstriction will decrease myocardial contractility. Whether NO has a direct role in the regulation of cardiac contractility independently of coronary vasoconstriction remains controversial, since it has been characterized as positive [35,36], absent [37], and negative [6] on basal myocardial contractility. Similar to the results here presented, in the rat isolated perfused heart, infusion of the heart with N^W^-nitro-L-arginine (L-NNA) provoked relevant decreases in both left ventricular pressure and dP/dtmax [38]. The infusion of another NOS inhibitor, N^G^-Methyl-L-arginine acetate salt (L-NMMA), in the rat isolated perfused heart caused an approximately 40% reduction in coronary flow and 60% in cardiac output [34]. In the rat isolated perfused heart stimulated with isoproterenol, infusion of L-NMA caused left ventricular dP/dt_max_ to decrease from 2718 ± 170 to 2070 ± 137 mm Hg/s^−1^ and left ventricular peak pressure to decrease from 105 ± 9 to 86 ± 5 mm Hg [39]. In vivo, intracoronary infusion of the NOS inhibitor L-NMMA (25 mmol/min) in healthy volunteers caused a significant reduction in basal LV dP/dt_max_ (from 1826 to 1578 mmHg/s; *p* < 0.002), but had no effect on mean aortic pressure or right atrial pressure, indicating that endogenous NO has a positive inotropic effect in the normal human heart [40]. The intravenous infusion of L-NMMA (1 mg/kg/min) caused an increase in the mean blood pressure and significant reductions in both cardiac output and stroke volume by 27.8 ± 2.9% and 15.4 ± 3.5%, respectively [41]. In Long Evans conscious rats, an intravenous bolus injection of L-NAME (10 mg kg^−1^) provoked important decreases in total cardiac output and stroke volume [42]. Interestingly, in a mouse model of heart failure with preserved ejection fraction, chronic administration of L-NAME did not alter the ejection fraction, although the animals presented signs of congestive heart failure. Indeed, acute or chronic administration of L-NAME is associated with cardio depressive effects [40,43,44,45,46,47].

Although the mechanism responsible for the inotropic action of 6-ND is not known, the finding that it also has positive chronotropic action may provide some clues. For instance, positive inotropic drugs increase the force of myocardial contraction through different pathways. The oldest, cardiac glycoside digoxin, works directly on myocardium and inhibits Na^+^/K^+^-ATPase in the cell membrane [48], resulting in an increase in the intracellular calcium content and its binding to contractile proteins of myofibril [49]. Istaroxime is a novel inotropic agent, structurally not related to cardiac glycosides but with a similar mechanism of action; it also inhibits Na^+^/K^+^-ATPase at the sarcolemma, increasing the intracellular calcium content during systole and improving contractility [50]. It is unlikely that 6-ND acts as an inhibitor of Na^+^/K^+^-ATPase since the use of digoxin is associated with a significant reduction in heart rate [51], and istaroxime, when administered (3 mg/kg/min) to normally conducted sinus rhythm dogs, decreased the heart rate [52].

Levosimendan, a pyridazinone-dinitrile derivative, is a positive inotropic drug with a different mechanism of action. It behaves as a calcium sensitizer, increasing troponin C affinity for Ca^2+^ and stabilizing the troponin C conformation [53]. Levosimendan can increase intracellular calcium due to phosphodiesterase inhibition; however, this does not occur in therapeutic concentrations [54]. The use of levosimendan in severe heart failure patients is associated with an improvement in haemodynamic functions without significant changes in heart rate [55]; while 6-ND has positive chronotropic action, the results obtained here show that 6-ND can have an inotropic effect at dose/concentrations that do not change the heart rate. Whether 6-ND can increase troponin C’s affinity for Ca^2+^ remains to be investigated.

Other positive inotropic agents increase cardiac contractility through pathways such as an increase in cyclic AMP levels either by stimulation of adenylyl cyclase or by inhibition of type-3 PDE [56]. The PDE3 inhibitors milrinone [57] and cilostazol [58] have positive inotropic and chronotropic effects, and the potency parallels their ability to inhibit PDE3. However, it is unlikely that 6-ND acts as a PDE3 inhibitor since, when incubated in the rat isolated right atrium, it abolishes the concentration-dependent increase in the atrial rate induced by milrinone or cilostazol [59]. In addition, incubation of 6-ND with human washed platelets was not accompanied by a change in nucleotide levels [60].

β_1_-receptor stimulation in ventricular myocytes activates the G_s_-adenylyl cyclase-cAMP-protein kinase A (PKA) with consequent phosphorylation of PKA substrates including L-type calcium channel, cardiac troponin I, and cardiac myosin-binding protein C, causing an increase in calcium transients and contractility [61]. In pacemaker cells, phosphorylation by PKA of membrane ion channels increases calcium cycling and pacing rate. Thus, the activation of β _1_-adrenoceptors by catecholamines is responsible for positive inotropy [62]. It is interesting that selective β_1_-adrenoceptor antagonists, atenolol, betaxolol, and metoprolol, reduced the atrial frequency at concentrations that selectively blocked the increases in atrial rate induced by 6-ND, indicating that the reduction in heart rate induced by β_1_-adrenoceptor antagonists could be due to specific inhibition of the 6-ND receptor, rather than the β_1_-adrenoceptor [19]. Indeed, the same phenomena has been observed here, where atenolol induced a negative inotropic effect at the same concentration that affected the positive inotropic effect of 6-ND but did not alter the positive inotropic effect induced by the classical catecholamines. The finding that atenolol presents a negative inotropic effect in concentrations that selectively affect the 6-ND positive inotropic effect reinforces the concept that 6-ND has a major modulatory role in heart inotropism.

Although the mechanism(s) by which 6-ND causes a positive inotropic and chronotropic effect in the rat isolated heart is unclear, the remarkable potency of this endogenous novel catecholamine presents interesting therapeutic possibilities, mainly in acute heart failure (AHF). This clinical syndrome, identified by a sudden worsening of AHF symptoms [63], is one of the most common causes for hospital admission, mainly in aged patients [64]. Guidelines recommend patients should receive temporary intravenous positive inotropic agents, such as adrenergic agonists, including dobutamine, dopamine, and PDE inhibitors such as milrinone [65]. Although there is some evidence that this approach maintains systemic perfusion and preserves end-organ performance, positive inotropic agents have not yet demonstrated improved outcomes in AHF patients in either a hospital or ambulatorial setting [66]. Interestingly, the most promising therapeutic target in acute decompensated AHF outside of decongestion with diuresis is the vasodilatory pathway [67], and 6-ND is also a very potent vasodilator [14,15]. However, it is important to note that, so far, the positive inotropic effect induced by 6-ND has been observed only in vitro, and therefore these results need to be validated in both anaesthetized and conscious animal models of heart failure.

## 5. Conclusions

6-ND has potent positive inotropic action, presenting a potential novel therapeutic approach in acute heart failure.

## Figures and Tables

**Table 1:** List of suppliers and chemicals.

Suppliers	Chemicals
Sigma-Aldrich Chemicals Co. (MO, USA)	Dopamine, adrenaline, and L-NAME (N^ω^-Nitro-L-arginine methyl ester hydrochloride)
Cayman Chemicals (MI, USA)	Noradrenaline and tetrodotoxin (TTX)
Toronto Research Chemicals (ON, Canada).	6-Nitrodopamine-d_4_ and 6-Nitrodopamine
CDN Isotopes (QC, Canada)	Adrenaline-d_6_ hydrochloride, DL-noradrenaline-d_6_ hydrochloride, and dopamine-d_3_ hydrochloride,
Merck KGaA (Hesse, Germany)	Sodium chloride (NaCl), dextrose, calcium chloride (CaCl_2_), magnesium sulfate (MgSO_4_), sodium bicarbonate (NaHCO_3_), potassium chloride (KCl), potassium phosphate monobasic (KH_2_PO_4_)

## Data Availability

Not applicable.
